# The Role of hCG Triggering Progesterone Levels: A Real-World Retrospective Cohort Study of More Than 8000 IVF/ICSI Cycles

**DOI:** 10.3389/fendo.2020.547684

**Published:** 2020-09-23

**Authors:** Raffaella De Cesare, Emanuela Morenghi, Federico Cirillo, Camilla Ronchetti, Valentina Canevisio, Paola Persico, Annamaria Baggiani, Maria Teresa Sandri, Paolo Emanuele Levi-Setti

**Affiliations:** ^1^Division of Gynecology and Reproductive Medicine, Department of Gynecology, Fertility Center, Humanitas Clinical and Research Center—IRCCS, Milan, Italy; ^2^Biostatistics Unit, Humanitas Clinical and Research Center, IRCCS, Milan, Italy; ^3^Clinical Analysis Laboratory, Humanitas Clinical and Research Center, IRCCS, Milan, Italy

**Keywords:** progesterone elevation, embryo transfer (ET), clinical pregnancy rate (CPR), live birth rate (LBR), serum progesterone levels

## Abstract

**Objective:** To assess the association between serum ovulation trigger progesterone (P) levels and the outcome of *in vitro* fertilization cycles.

**Design Setting:** Real world single-center retrospective cohort study.

**Patient Intervention(s):** All fresh cleavage and blastocyst-stage embryo transfers (ETs) performed from January 2012 to December 2016.

**Main outcome Measure(s):** The impact of premature high serum P levels cycles in terms of clinical pregnancy rates (CPRs) and live birth rates (LBRs).

**Results:** 8,034 ETs were performed: 7,597 cleavage-stage transfers and 437 blastocyst transfers. Serum P levels demonstrated to be inversely related to CPR (OR 0.72, *p* < 0.001) and LBR (OR 0.73, *p* < 0.001). The progressive decrease of LBR and CPR started when P levels were >1 ng/ml in a good prognosis cleavage ET subgroup, whereas in patients with worse prognosis only for *P* ≥ 1.75 ng/ml. In the blastocyst ET subgroup, the negative effect of P elevation was reported only if P was >1.75 ng/ml. CPR (OR 0.71 (0.62–0.80), *p* < 0.001) and LBR (OR 0.73 (0.63–0.84), *p* < 0.001) in thawed cycles resulted statistically significantly higher than in fresh cycles in the cleavage-stage subgroup. In the blastocyst group, no significant difference resulted between thawed and fresh cycles, independently of P levels [CPR OR 0. 37 (0.49–1.09), *p* = 0.123; LBR OR 0.71 (0.46–1.10), *p* = 0.126].

**Conclusion:** High P levels decrease CPR as well as LBR in both cleavage and blastocyst ET. In the cleavage group, for P levels below 1.75 ng/ml, our data suggest the possibility to wait until day 5 for ET, and if P level is ≥1.75 ng/ml, it should be considered to freeze all embryos and postpone the ET.

**Clinical Trial Registration:** ClinicalTrials.gov, ID: NCT04253470

## Introduction

The association between serum progesterone (P) levels, measured on the day of ovulation trigger, and the outcome of *in vitro* fertilization (IVF) cycles, has been one of the major controversies in the field of ovarian stimulation endocrinology (1, 2). Since 1991, many studies have emphasized that a premature and excessive P increase just before the induction of ovulation might negatively affect the outcome of the IVF cycle (3–6). However, a good number of publications have similarly reported opposite conclusions (7–10). Until now, the cause of P increase is not clearly known. The high doses of exogenous follicular stimulating hormone (FSH) used in ovarian stimulation cycles could cause the rise of P levels toward the end of the follicular phase. FSH directly induces the enzymatic activity of 3-beta-hydroxysteroid dehydrogenase and thus increases the conversion of pregnenolone to P. Moreover, low levels of luteinizing hormone (LH) could reduce the transformation of pregnenolone and P into androgens by theca cells (11). Some authors report that a premature increase of P during ovarian stimulation could be caused by a loss of activity of LH and human chorionic gonadotropins (hCGs) (12, 13).

The association between an excessive elevation of P and a negative outcome of the IVF cycle has been the subject of a still ongoing debate. A large meta-analysis, counting more than 55,000 cycles, has provided convincing data supporting the hypothesis that the elevation of serum P is associated with a lower pregnancy rate (14). However, an extensive review of the literature, rather than providing a final answer on the role of P on pregnancy probability, has generated new important clinical questions. Is there a P threshold that can predict IVF cycle outcome? Can the transfer of embryos at the blastocyst stage reverse the negative impact that high P levels may have? Might the use of gonadotropin-releasing hormone (GnRH) agonist or antagonist protocol change the levels of progesterone? Are the effects of high P levels the same for all patients, independently of their characteristics and their answer to ovarian stimulation?

The aim of this study is to review the dataset of a single university-affiliated center to evaluate the impact of premature high serum P levels on the outcome of fresh embryo transfer cycles in terms of clinical pregnancy rates (CPRs) and live birth rates (LBRs). Over the years, literature has considered different progesterone cut-off levels that have been analyzed in the present real-world data (15) to assess their appropriateness and feasibility in our population to detect their eventual different impact on different patients' categories or on different stages of embryo maturation. In fact, furthermore, the present study retrospectively investigates whether the transfer of blastocysts on day 5 post-fertilization (D5-ET) may improve the CPR and the LBR in patients with P rise.

## Materials and Methods

A single-center retrospective cohort study was performed in a tertiary-care academic fertility center (Humanitas Fertility Center, Rozzano, Milan, Italy).

All fresh embryo transfers both at cleavage stage (day 2 and day 3) and blastocyst stage (day 5), performed from January 2012 to December 2016, were included.

The following clinical variables were analyzed: age, smoking habit, body mass index (BMI), causes of infertility, time they have been trying to conceive, and ovarian stimulation protocols. Other measured parameters included basal FSH and anti-Mullerian hormone (AMH) levels, estradiol (E2) and P levels on the day of ovulation induction, number of retrieved oocytes, embryo stage on the day of transfer, and number of transferred embryos.

The controlled ovarian stimulation (COS) protocol provided the use of recombinant FSH (rFSH), hMG, or rFSH + recombinant LH (rFSH + rLH). The gonadotropin starting dose was determined according to ovarian reserve parameters, such as AMH, antral follicle count (AFC), and BMI. COS was performed using four different protocols: GnRH agonist long protocol; GnRH agonist short protocol; GnRH antagonist protocol; flare-up GnRH agonist protocol. Most of the antagonist COS started with the use of combined oral contraceptive pretreatment.

The COS protocol was chosen based on patients' characteristics and gynecologists' judgment, according mainly to Poseidon criteria (16–18).

Long GnRH agonist protocol was based on the administration of daily leuprorelin (Enantone die; Takeda, Italy) or leuprolin acetate 0.1 mg/day (Fertipeptyl; Abbott Pharmaceutical Products, USA) on day 21 of the previous luteal phase of the stimulation cycle. When pituitary desensitization was achieved (14 days after the initiation of GnRH agonist), as evidenced by the absence of ovarian follicles >5 mm and endometrial thickness <5.4 mm on transvaginal ultrasound examination, gonadotropin stimulation was initiated. In short GnRH agonist protocol, the agonist (leuprolin 0.1 mg/day) was administered from day 21 of the previous cycle and induction from day 1 or 2 of the cycle (day 1 being the start of the menstrual bleed) reducing the agonist dose to 0.05 mg/day and continuing with stimulation until the day of hCG administration. In the GnRH antagonist protocol, on the first day of women spontaneous menstrual cycle or a withdrawal bleeding after receiving a low-dose oral contraceptive, gonadotropin stimulation was initiated, and when the leading follicle reached 13–14 mm in mean diameter and/or plasma E2 exceeded 400 pg/ml, an injection of 0.25 mg of GnRH antagonist (Cetrotide, Merck Serono S.p.A., Rome, Italy; Orgalutran, Organon, MSD-Italy) was administered by a subcutaneous (sc) injection daily until the day of ovulation trigger.

Finally, in the flare-up GnRH protocol, daily agonist was provided on day 1 of the cycle with triptorelin (0.1 mg/day) and then gonadotropins were started according to ovarian reserve parameter on day 2 of the cycle. A starting variable dose of gonadotropin [hMG (Meropur; Ferring, Milan, Italy) or rFSH (Puregon, MSD-Italy; Gonal-F, Merck Serono S.p.A., Rome, Italy)] with or without the addition of r-LH for the first 4 days and then an individualized dose were administered according to the parameters resulting from transvaginal ultrasound and estradiol and progesterone levels until the day of ovulation trigger. The protocol of induction and the dose of gonadotropins administered were tailored on an individual basis according to the patient's age, serum hormonal levels, and AFC. Transvaginal ultrasonography, estradiol, and progesterone determinations were performed during COS. When at least three follicles with a mean diameter >17 mm were observed, 250 μg of recombinant hCG (Ovitrelle; Merck Serono S.p.A.) was administered subcutaneously. Oocyte retrieval was performed transvaginally 36 h after hCG injection. Embryo transfer was performed from days 3 to 5 after oocyte collection. Luteal phase was supported in all patients with vaginal progesterone (Crinone 8% twice a day, Merck Serono S.p.A.; or Prometrium, Rottapharm 600 mg a day) starting the evening of oocyte retrieval and continued in pregnant patients until the ninth gestational week.

In high responder (HR) patients (more than 18 follicles with diameter ≥12 mm at ovulation induction), the trigger was obtained with triptorelin 0.2 mg sc. In this cohort, if <18 oocytes were retrieved, patients were considered at intermediate risk of ovarian hyperstimulation syndrome (OHSS) and fresh transfer was performed. An adequate support of the luteal phase was initiated: hCG 1,500 IU/sc the day of the retrieval + estradiol 4 mg + vaginal progesterone 400 mg daily (rescue protocol).

Serum hCG was assessed 2 weeks after embryo transfer and then every 48 h until a value over 1,000 mIU was detected and a vaginal ultrasound was scheduled 4 weeks after the embryo transfer.

The ARCHITECT Estradiol assay (Abbott, Ireland, Diagnostics Division, Longford, Ireland) is a chemiluminescent microparticle immunoassay (CMIA) for the quantitative determination of estradiol in human serum and plasma. The analytical sensitivity of the ARCHITECT Estradiol assay is ≤ 10 pg/ml. The functional sensitivity of the ARCHITECT Estradiol assay is ≤ 25 pg/ml. The ARCHITECT Progesterone assay (Abbott, Ireland, Diagnostics Division, Longford, Ireland) is a CMIA for the quantitative determination of progesterone in human serum and plasma. The ARCHITECT Progesterone assay is designed to have an analytical sensitivity of ≤ 0.1 ng/ml. This assay is designed to have a precision of ≤ 10% total inter- and intra-assay CV (coefficient of variation) for concentrations in the range of the ARCHITECT Progesterone Low Control.

On day 3 after fertilization, the number of transferred embryos varied depending on age, prognosis, results of previous IVF cycles, and on gynecological and obstetrical history.

In patients showing good prognostic features (i.e., age <39 years, more than 4 zygotes on day 1 post-fertilization, good-quality ejaculated sperm sample), the transfer of a single blastocyst-stage embryo was performed. In patients with *P* >3 ng/ml, a freeze-all strategy was chosen (19).

Progesterone results were stratified in four groups: <1, 1–1.5, 1.5–1.75, and ≥1.75.

A general freeze-all policy was decided for P elevation over 1.5 ng/ml even if a cut-off limit was properly introduced in our protocols only from January 2018, when our preliminary data showed a clear tendency to reduce pregnancy rate even if a reduction was already present at 1.0 ng/ml.

We also compared CPR and LBR with thawed cycles from the same period, excluding the ones deriving from PGTA (20).

For the purposes of this study, our population was further divided into four groups based on age and number of retrieved oocytes:

Age ≤ 38, oocyte ≥8.
Age ≤ 38, oocyte <8.
Age >38, oocyte ≥8.
Age >38, oocyte <8.

Frozen embryo cycles (FET) protocol: in natural cycles, patients had serial transvaginal ultrasound monitoring (TU) starting between cycle days 8 and 12 to detect the dominant follicle and assessing endometrial development. Patients were instructed to start monitoring for urinary LH testing when a follicle with a mean diameter >11 mm was identified. The LH testing was carried out in the early morning before the TU. When the endometrial thickness reached 7 mm and the dominant follicle was 16–20 mm in diameter, patients were considered ready for planning embryo transfer. In patients with no positive urinary LH test despite a follicle of 16–20 mm and endometrial stripe of 7 mm or more, 5,000 U of urinary hCG (Gonasi HP, Ibsa, Italy) were administered. Embryo rewarming and transfer was planned 7 days after the spontaneous LH peak or HCG administration.

Hormonal replacement cycles (AR-FET) consisted of oral estradiol valerate (E2V, 6 mg) (Progynova; Bayer, Schweiz, AG, 2 mg) from the second day of the menstrual cycle until the endometrial thickness reached at least 7 mm. The embryo transfer was scheduled after 3–5 days from the progesterone start, continuing the same estradiol dose. If endometrial thickness was less than 7 mm after 12 days of E2V, the dose was increased to 8 mg/day. Endometrial preparation for transfer consisted of continued estradiol (6–8 mg a day E2V) combined with 600 mg of vaginal micronized progesterone tablets (Prometrium, Rottapharm S.p.A., or Progeffik, Effik Italia S.p.A., 200 mg every 8 h) or 180 mg of micronized progesterone vaginal gel (Crinone 8%, Merck, Serono, 90 mg twice a day). Exogenous progesterone supplementation was also started on the day of embryo transfer in the NC-FET group and 2 days after hCG administration in the mNC-FET group using 200 mg vaginal micronized progesterone tablets (Prometrium, Rottapharm S.p.a., or Progeffik, Effik Italia S.p.a., 200 mg every day) or 90 mg micronized progesterone vaginal gel (Crinone 8%, Merck, Serono, 90 mg once a day).

Pregnancy tests (serum beta-hCG) were obtained 12 after ET, and if positive, beta-hCG levels were monitored every 48 h until they reached at least 1,000 IU/ml. Transvaginal US was performed 2 weeks later to determine the number of gestational sacs and fetal viability.

Patients continued progesterone supplementation and estradiol in AR-FET until week 12 of gestation.

### Statistical Analysis and Variable Description

Clinical pregnancy was defined as indicated by WHO-ICMART Consensus and the ESHRE register: a pregnancy diagnosed by ultrasonographic visualization of one or more gestational sacs or definitive clinical signs of pregnancy (21).

### Statistical Analysis and Consideration About Data Storage

All data were first recorded in the digitalized medical charts routinely in use at the Humanitas Fertility Center; all databases are protected by a password. Thereafter, the data of highest interest were moved to another database for better management and easier elaboration.

The investigators guarantee that this study respected the Declaration of Helsinki (22) and the national Italian law in terms of the respect of patient data protection (23). All patients signed an informed consent form before gaining access to the Assisted Reproduction Technology (ART) program of our center; this document specifically allows the treatment of personal data for retrospective studies.

The present study obtained the definitive approval by Humanitas Institutional Review Board (IRB) on January 2020 and was registered, in accordance with International Committee of Medical Journal Editors, in ClinicalTrials.gov, registry (ID NCT04253470).

Pregnancies were defined according to international glossary (21) and more in detail in our center; clinical pregnancy was defined as a positive beta-hCG test ≥1,000 mIU/ml, while live births consisted in all the births of a live fetus occurred after the 24th week of gestation.

All data were expressed as number and percentage, or as mean and SD. The impact of P level on CPR and LBR was explored using a multivariable logistic regression. The possible confounding variables were chosen based on the daily clinical practice and the literature and not on the previously performed univariate analysis and *p*-values. A *p*-value lower than 0.05 was considered significant. All analyses were carried out using Stata 15.0 (StataCorp. 2017. Stata Statistical Software: Release 15. College Station, TX: StataCorp LP).

## Results

During the study period, 8,034 embryo transfers were performed: 7,597 cleavage-stage transfers (D3) and 437 blastocyst transfers (D5). We also included 3,112 thawing cycles, of whom 2003 (64.36%) resulted by supernumerary embryo cycles, 107 (3.44%) by P increase, 796 (25.58%) by OHSS, and 206 (6.62%) by other reasons (i.e., inadequate endometrium, medical complications, etc).

Baseline characteristics and comparison between cleavage-stage and blastocyst-stage populations showed statistically significant differences in woman age (37.1 ± 4.0 vs. 35.7 ± 4.0, *p* < 0.001), BMI (22.25 ± 3.30 vs. 21.61 ± 2.95, *p* < 0.001), FSH (7.66 ± 3.03 vs. 6.76 ± 2.03, *p* < 0.001), AMH (2.18 ± 3.60 vs. 3.07 ± 2.70, *p* < 0.001), reduced ovarian reserve diagnosis [1,417 (18.65%) vs. 20 (4.58%), *p* < 0.001], progesterone on trigger day (0.88 ± 0.45 vs. 1.09 ± 0.54, *p* < 0.001), number of retrieved oocytes (8.31 ± 4.48 vs. 12.91 ± 4.38, *p* < 0.001), mature oocytes (7.33 ± 4.21 vs. 11.54 ± 4.15, *p* < 0.001), fertilized oocytes (4.03 ± 2.22 vs. 6.68 ± 1.79, *p* < 0.001) as in the number of transferred embryos, frozen embryos, and frozen oocytes (Table 1).

**Table 1:** Baseline characteristics.

	**All embryo transfers**	**Day 3 embryo transfers**	**Day 5 embryo transfers**	***p***
Number of embryo transfers	8,034	7,597	437	
Patient's age	37.0 ± 4.0	37.1 ± 4.0	35.7 ± 4.0	<0.001
Active smoking	1,581 (19.68%)	1,503 (19.78%)	78 (17.85%)	0.322
Body mass index	22.22 ± 3.29	22.25 ± 3.30	21.61 ± 2.95	<0.001
**Infertility causes**				
Male factor	2,623 (32.65%)	2,441 (32.13%)	182 (41.65%)	<0.001
Tubal factor	779 (9.24%)	702 (9.24%)	77 (17.62%)	<0.001
Idiopathic	767 (9.55%)	713 (9.39%)	54 (12.36%)	0.040
Mixed male and female factors	1,557 (19.38%)	1,497 (19.71%)	60 (13.73%)	0.002
Endometriosis	390 (4.85%)	362 (4.77%)	28 (6.41%)	0.120
Reduced ovarian reserve	1,437 (17.89%)	1,417 (18.65%)	20 (4.58%)	<0.001
Anovulatory	101 (1.26%)	96 (1.26%)	5 (1.14%)	1.000
Multiple female factor	363 (4.52%)	354 (4.66%)	9 (2.06%)	0.009
Repeated pregnancy failure	17 (0.21%)	15 (0.20%)	2 (0.46%)	0.236
Basal follicle-stimulating hormone	7.61 ± 2.99	7.66 ± 3.03	6.76 ± 2.03	<0.001
Basal anti-Mullerian hormone	2.23 ± 3.56	2.18 ± 3.60	3.07 ± 2.70	<0.001
Stimulation protocol (antagonist)	4,538 (56.48%)	4,304 (56.65%)	234 (53.55%)	0.203
Progesterone on trigger day	0.90 ± 0.45	0.88 ± 0.45	1.09 ± 0.54	<0.001
Intracytoplasmic sperm injection (ICSI)	7,642 (95.12%)	7,226 (95.12%)	416 (95.12%)	0.941
Retrieved oocytes	8.56 ± 4.60	8.31 ± 4.48	12.91 ± 4.38	<0.001
Mature oocytes	7.56 ± 4.31	7.33 ± 4.21	11.54 ± 4.15	<0.001
Injected oocytes	5.79 ± 2.75	5.64 ± 2.71	8.46 ± 2.05	<0.001
Fertilized oocytes	4.17 ± 2.28	4.03 ± 2.22	6.68 ± 1.79	<0.001
Transferred embryos	2.10 ± 0.62	2.12 ± 0.62	1.73 ± 0.44	<0.001
Frozen embryos	0.51 ± 0.96	0.47 ± 0.92	1.16 ± 1.34	<0.001
Frozen oocytes	0.42 ± 1.72	0.39 ± 1.67	0.95 ± 2.49	<0.001

The use of recombinant LH or FSH, HMG, the type of protocol (agonist vs. antagonist), the choice of rescue cycles with agonist triggering, or the pre-treatment taking of oral contraceptive pills as other variables were considered in a preliminary univariate assay, but results were not confirmed in the consequent multivariate analysis in which no difference was depicted among the different groups.

Analyzing progesterone elevation in relation to different induction protocols was not in the principal and secondary outcome in the originally approved and submitted protocol, and sample size was not considered for these variables [Mol, Bossuyt, Sunkara, Garcia Velasco, Venetis, Sakkas, Lundin, Simón, Taylor, Wan, Longobardi, (24, 25)], and for these reasons, no further analysis was conducted.

After adjustment for maternal age, and number of retrieved oocytes and transferred embryos, serum P levels demonstrated to be inversely related to CPR and LBR, which decreased when the serum P levels were >1 ng/ml [0.72 (0.63–0.81), *p* < 0.001 and 0.73 (0.64–0.84), *p* < 0.001]. For progesterone level between 1 and 1.5 ng/ml, the OR for CPR was 0.85 (0.76–0.96), *p* = 0.008 and for LBR was 0.85 (0.75–0.97), *p* = 0.016. A further decrease was observed with P levels >1.75 ng/ml resulting in a CPO OR of 0.55 (0.43–0.71), *p* < 0.001 and LBR OR of 0.55 (0.42–0.73), *p* < 0.001 (Table 2).

**Table 2:** Influence of progesterone level on clinical pregnancy rate (CPR) and live birth rate (LBR).

	**CPR**		**LBR**	
	**OR (95% CI)**	***p***	**OR (95% CI)**	***p***
Progesterone level	0.72 (0.63–0.81)	<0.001	0.73 (0.64–0.84)	<0.001
<1	1		1	
1–1.5	0.85 (0.75–0.96)	0.008	0.85 (0.74–0.97)	0.017
1.5–1.75	0.69 (0.55–0.85)	0.001	0.77 (0.61–0.97)	0.025
≥1.75	0.55 (0.43–0.71)	<0.001	0.55 (0.41–0.73)	<0.001
**Day 3**				
Progesterone level	0.71 (0.62–0.80)	<0.001	0.73 (0.64–0.83)	<0.001
<1	1		1	
1–1.5	0.87 (0.77–0.99)	0.033	0.87 (0.76–1.00)	0.051
1.5–1.75	0.65 (0.52–0.82)	<0.001	0.71 (0.56–0.91)	0.008
≥1.75	0.53 (0.41–0.70)	<0.001	0.55 (0.40–0.74)	<0.001
**Day 5**				
Progesterone level	0.73 (0.50–1.08)	0.118	0.71 (0.47–1.09)	0.117
<1	1		1	
1–1.5	0.60 (0.37–0.96)	0.034	0.61 (0.36–1.02)	0.061
1.5–1.75	0.95 (0.49–1.85)	0.877	1.24 (0.62–2.48)	0.534
≥1.75	0.52 (0.26–1.03)	0.062	0.45 (0.21–1.01)	0.052

*All the results are corrected for age, number of retrieved oocytes, and number of transferred embryos*.

The rates were analyzed also considering the day of embryo transfer. In the D3 group, a serum P level ≥1 ng/ml significantly reduced CPR and LBR resulting in an OR of 0.71 (0.62–0.80), *p* < 0.001 and 0.73 (0.63–0.84), *p* < 0.001, respectively. For progesterone levels between 1 and 1.5 ng/ml, the OR was 0.87 (0.77–0.99), *p* = 0.030 for CPR and 0.87 (0.76–1.00), *p* = 0.048 for LBR. With a P level ≥1.75 ng/ml, CPR and LBR decreased even more to nearly half their value with an OR of 0.53 (0.41–0.70), *p* ≤ 0.001 and 0.55 (0.40–0.74), *p* < 0.001.

The relationship between serum P level and CPR and LBR in the D5 group instead was not significantly different [OR 0.37 (0.49–1.09), *p* = 0.123 and 0.71 (0.46–1.10), *p* = 0.126]. For progesterone values between 1 and 1.5 ng/ml, the OR was 0.60 (0.37–0.96), *p* = 0.034 for CPR and 0.61 (0.36–1.02), *p* = 0.061 for LBR. In the progesterone values group between 1.5 and 1.75 ng/ml, the OR was 0.95 (0.49–1.85), *p* = 0.877 for CPR and 1.24 (0.63–2.47), *p* = 0.533 for LBR. When the values were ≥1.75 ng/ml, the OR was 0.52 (0.26–1.03), *p* = 0.062 for CPR and 0.45 (0.21–0.99), *p* = 0.047 for LBR. These results are reported in Table 2 as well.

To evaluate eventual differences in CPR depending on different P thresholds, the analysis was subsequently performed separately in different subgroups.

In the 2,555 D3 good prognosis embryo transfers subgroup (i.e., women aged ≤ 38 years and number of oocytes retrieved ≥8), the CPR was 38.6% and the LBR was 32.2% for *P* < 1 ng/ml. The effect of P increase to values between 1 and 1.5 ng/ml resulted in a 34.7% CPR [OR 0.84 (0.70–1.01), *p* = 0.061] and a 28.4% LBR [OR 0.83 (0.69–1.00), *p* = 0.055]. For *P* > 1.5 ng/ml, the CPR was 28.5% [OR 0.63 (0.47–0.85), *p* = 0.003] and 25.2% [OR 0.71 (0.52–0.97), *p* = 0.031] for LBR. In the ≥1.75 P group, the CPR was 29.4% [OR 0.66 (0.47–0.92), *p* = 0.015] and the LBR was 25.0% [OR 0.69 (0.49–0.99), *p* = 0.046] (Table 3).

**Table 3:** D3 embryo transfer subgroup: influence of P levels on clinical pregnancy rate (CPR) and live birth rate (LBR), stratified by woman age and number of retrieved oocytes and corrected for number of embryo transfer.

			**CPR**			**LBR**	
**Progesterone level**	***N***	***n* (%)**	**OR (95% CI)**	***p***	***n* (%)**	**OR (95% CI)**	***P***
Age ≤ 38, oocyte ≥8	2,555						
<1	1,281	495 (38.6)	1		413 (32.2)	1	
1–1.5	852	296 (34.7)	0.84 (0.70–1.01)	0.065	242 (28.4)	0.83 (0.69–1.01)	0.057
1.5–1.75	242	69 (28.5)	0.63 (0.47–0.86)	0.003	61 (25.2)	0.71 (0.52–0.97)	0.031
≥1.75	180	53 (29.4)	0.66 (0.47–0.92)	0.015	45 (25.0)	0.69 (0.49–0.99)	0.046
Age ≤ 38, oocyte <8	1,964						
<1	1,470	391 (26.6)	1		325 (22.1)	1	
1–1.5	369	94 (25.5)	0.92 (0.71–1.20)	0.539	75 (20.3)	0.88 (0.66–1.17)	0.377
1.5–1.75	79	19 (24.1)	0.85 (0.50–1.45)	0.557	15 (19.0)	0.81 (0.45–1.44)	0.471
≥1.75	46	9 (19.6)	0.63 (0.30–1.32)	0.221	5 (10.9)	0.40 (0.16–1.03)	0.058
Age >38, oocyte ≥8	1,266						
<1	598	179 (29.9)	1		109 (18.2)	1	
1–1.5	463	121 (26.1)	0.82 (0.62–1.08)	0.157	69 (14.9)	0.78 (0.56–1.08)	0.138
1.5–1.75	117	24 (20.5)	0.58 (0.36–0.96)	0.033	13 (11.1)	0.55 (0.30–1.01)	0.055
≥1.75	88	8 (9.1)	0.22 (0.11–0.48)	<0.001	4 (4.6)	0.21 (0.07–0.58)	0.003
Age >38, oocyte <8	1812						
<1	1,387	220 (15.9)	1		132 (9.5)	1	
1–1.5	328	49 (14.9)	0.84 (0.60–1.17)	0.301	34 (10.4)	0.99 (0.66–1.48)	0.950
1.5–1.75	51	4 (7.8)	0.38 (0.14–1.05)	0.062	3 (5.9)	0.50 (0.16–1.61)	0.246
≥1.75	46	5 (10.9)	0.64 (0.25–1.66)	0.358	2 (4.4)	0.43 (0.10–1.81)	0.248

In the 1964 age ≤ 38, oocyte <8 group of patients, the overall CPR was 26.6% and the LBR was 22.1% for *P* < 1 ng/ml, and if P was between 1 and 1.5 ng/ml, the CPR was 25.5% (*p* = 0.541) and the LBR was 20.3% (*p* = 0.374). In the 1.5–1.75 P group, CPR was 24.1% (*p* = 0.85) and the LBR was 19.0% (*p* = 0.469) and in the ≥1.75 group 19.6% (*p* = 0.63) and 10.9% (*p* = 0.058), respectively.

In the 1,266 age >38, oocyte ≥8 group, the CPR was 29.9% and the LBR was 18.2% for *P* < 1 ng/ml, and if P was between 1 and 1.5 ng/ml, the CPR was 26.1% (*p* = 0.147) and the LBR was 14.9% (*p* = 0.125). In the 1.5–1.75 P group, CPR was 20.6% (*p* = 0.030) and the LBR was 11.1% (*p* = 0.054) and in the ≥1.75 group 9.1% (*p* ≤ 0.001) and 4.6% (*p* = 0.003), respectively.

In the 1812 age >38, oocyte <8 group, the CPR was 15.9% and the LBR was 9.5% for *P* < 1 ng/ml, and if P was between 1 and 1.5 ng/ml, the CPR was 14.9% (*p* = 0.305) and the LBR was 10.4% (*p* = 0.950). In the 1.5–1.75 P group, CPR was 7.8% (*p* = 0.067) and the LBR was 5.9% (*p* = 0.354) and in the ≥1.75 group 10.9% (*p* ≤ 0.001) and 4.4.% (*p* = 0.244), respectively (Table 3).

In the D5 embryo transfers subgroup, the negative effect of P elevation on LBR and CPR was reported only if P was >1.75 ng/ml, independently of woman age or number of retrieved oocytes. This could depend on the fact that the size of each subgroup was not enough to guarantee sufficient statistical power to evaluate such differences. For this reason, results concerning this subgroup were not reported in any table.

In Table 4, the influence of P levels on CPR and LBR, reported as OR corrected by woman age, number of retrieved oocytes, and number of transferred embryos, is shown in women who underwent fresh embryo transfer, both in D3 and D5 groups, and those who underwent frozen-thawed cycles (independently of D3 or D5 embryos). CPR and LBR in thawed cycles resulted statistically significantly higher than in fresh cycles. Considering the D3 group, the thawed cycles group resulted with a better pregnancy outcome, both as CPR and LBR, even compared with patients with P level <1 ng/ml. In the D5 group, no significant difference resulted between thawed and fresh cycles, independently of P levels.

**Table 4:** Comparison between women who underwent fresh embryo transfer, both in day 3 (D3) and day 5 (D5) groups, and those who underwent frozen-thawed cycles (independently of D3 or D5 embryos): influence of P levels on clinical pregnancy rate (CPR) and live birth rate (LBR) (OR are corrected by woman age, number of retrieved oocytes, and number of transferred embryos).

			**CPR**			**LBR**	
**Progesterone level**	***N***	***n* (%)**	**OR (95% CI)**	***p***	***n* (%)**	**OR (95% CI)**	***p***
<1	4,941	1,369 (27.7)	1		1,043 (21.1)	1	
1–1.5	2,145	601 (28.0)	0.92 (0.82–1.03)	0.144	450 (21.0)	0.91 (0.80–1.03)	0.127
≥1.5	948	226 (23.8)	0.70 (0.59–0.83)	<0.001	175 (18.5)	0.73 (0.61–0.88)	0.001
Thawing cycle	3,242	1,169 (36.1)	1.34 (1.18–1.52)	<0.001	887 (27.4)	1.21 (1.05–1.38)	0.007
**Day 3**							
<1	4,736	1,285 (27.1)	1		979 (20.7)	1	
1–1.5	2,012	560 (27.8)	0.94 (0.83–1.06)	0.302	420 (20.9)	0.93 (0.81–1.06)	0.257
≥1.5	849	191 (22.5)	0.67 (0.56–0.80)	<0.001	148 (17.4)	0.70 (0.58–0.85)	<0.001
Thawing cycle	3,242	1,169 (36.1)	1.42 (1.24–1.61)	<0.001	887 (27.4)	1.26 (1.09–1.45)	0.002
**Day 5**							
<1	205	84 (41.0)	1		64 (31.2)	1	
1–1.5	133	41 (30.8)	0.65 (0.41–1.03)	0.067	30 (22.6)	0.65 (0.39–1.08)	0.095
≥1.5	99	35 (35.4)	0.81 (0.50–1.35)	0.427	27 (26.3)	0.87 (0.51–1.48)	0.600
Thawing cycle	3,242	1,169 (36.1)	0.79 (0.59–1.06)	0.121	887 (27.4)	0.80 (0.59–1.10)	0.175

## Discussion

The results of the present study show that high P levels, measured on the ovulation-trigger day, decrease CPR as well as LBR in both D3 and D5 fresh embryo transfers, as reported by the literature (14, 26, 27).

The precise mechanism explaining the effect that high P levels occurring on the day of hCG administration may have on the IVF cycle outcome is still unclear. The quality of oocytes (27) and the endometrial receptivity play a substantial role, but the role of progesterone elevation in oocyte quality, maturity, and ovulation is controversial (28, 29), as shown in studies on endometrial gene expression (30, 31) as well as in studies comparing the live birth rates of fresh cycles versus donor/recipient cycles or frozen-thawed embryo cycles (14, 32). The previously cited studies show that P elevation on the day of hCG administration is associated with a decreased probability of pregnancy achievement in fresh IVF cycles, but there is no statistically significant difference between cycles with high P levels and those without regarding frozen-thawed or donor cycles. It suggests that pre-hCG P elevation may affect endometrial receptivity and not embryo quality. In 1997, Ubaldi et al. (33) demonstrated that P elevation may modify the implantation rate depending on the timing at which it occurs. If it is delayed by more than 3 days, the asynchrony between endometrium receptivity and the embryo stage could decrease the implantation rate. This conclusion could be explained by the hypothesis that P elevation could advance the endometrial maturation.

The negative impact of P level elevation, which possibly depends on its effect on the endometrium, has been already assessed by previous studies that did not show P influence on the oocyte or embryo quality (34, 35). The same conclusion was reached by other studies on oocyte-donation cycles in which there was no evidence of a negative effect of the donor's P level at the end of the stimulation cycle (36).

As reported by recent studies and a meta-analysis (14, 27), the negative effect of P has been observed for levels >1 ng/ml, especially in cleavage embryo transfers (D3). Consequently, these authors recommended keeping P levels low to avoid a negative effect on fresh cycles. Different strategies are reported in the literature for this purpose, e.g., the use of a mild ovarian stimulation to maintain low estrogen levels (37, 38) or the induction of an earlier ovulation in HR patients (39) as well as in patients who had an early P elevation in an eventual previous cycle.

In our dataset, an elevation of progesterone levels (>1 ng/ml) is reported in over one third of our population (3092/8034 patients who underwent fresh cycles during the study period). Interestingly, a different impact of P level elevation was observed in different patient categories. In the D3 embryo transfer group, generally any serum P level ≥1 ng/ml significantly reduced both CPR and LBR. However, in patients with worse prognosis (number of retrieved oocytes <8), the effect of P increase was found to be not different, even considering woman age. It must be considered that in this category of patients, their worse outcome may depend on multiple factors, especially oocyte and embryo quality, while endometrial receptivity may play a secondary role.

The relationship between high serum P levels and both low CPR and LBR in the D5 embryo transfer group was not strongly significantly different even for serum P level exceeding the threshold of 1.75 ng/ml.

## Conclusions

From our study results, it appears that a blastocyst-stage embryo transfer allows setting the P threshold to <1.75 ng/ml to avoid P negative effect on CPR and LBR, but it does not permit to overcome the issue completely.

In the D3 group, for P levels below 1.75 ng/ml, it could be considered on our results to wait, if clinically possible, until D5 for embryo transfer, and if P level is ≥1.75 ng/ml, it should be considered to freeze all embryos and postpone the embryo transfer until P levels normalize, analyzing the center vitrification and warming results to support this choice.

CPR and LBR in thawed cycles resulted in our experience statistically significantly higher than in fresh cycles. Considering the D3 group, the thawed cycles group showed a better pregnancy outcome, both as CPR and LBR, even compared with patients with P level <1 ng/ml. In the D5 group, no significant difference resulted between thawed and fresh cycles, independently of P levels. On the other hand, it must be considered a possible bias that no P level measurements were carried out in women on the day of ovulation trigger by LH, hCG, or progesterone administration before embryo thawing that could be investigated in future studies because progesterone is considered a physiological trigger of ovulation (28).

From our analysis, embryo cryopreservation emerges as an optimal strategy to minimize the adverse effects of P elevation, especially if vitrified at blastocyst stage, and the importance of an efficient freezing program in cumulative delivery rate has been recently outlined (40). All centers need a strict analysis of their KPI such as blastocyst rate, warming survival rate, and fresh and post-warming implantation and evolutive pregnancy rate when deciding a freeze-all strategy in borderline progesterone elevations (41, 42). However, it is important to consider that this procedure cannot be carried out in all patients because their embryo transfer cannot always be granted because embryos may not reach the blastocyst stage to be vitrified or may not survive during the thawing procedure. Even in the best settings, survival rate is not 100% and could also be possible that prolonged embryo culture could be stressful for the embryos in some small subset of patients more than in others.

No differences related to the type of induction protocol were found, and minor but significant differences were found in subgroups of patients with good prognosis vs. worse prognosis. Our sample size was insufficient, however, to analyze other subset groups of patients, and other data are needed to further support evidences in this area.

In conclusion, progesterone elevation effect is evident in our results starting from values >1 ng/ml and significantly has a progressive influence on CPR and LBR in cleavage-stage transfer, but these effects become evident in blastocyst-stage transfers only for values >1.75 ng/ml.

## Data Availability Statement

The raw data supporting the conclusions of this article will be made available by the authors, without undue reservation.

## Ethics Statement

The studies involving human participants were reviewed and approved by Humanitas Ethics Board. The patients/participants provided their written informed consent to participate in this study.

## Author Contributions

PL-S and RD wrote the research project. EM analyzed data. FC, VC, CR, PP, AB, and MS contributed to data extraction, references and follow up analysis. FC and CR contributed to the manuscript preparation. PL-S prepared the final draft. All authors contributed to the article and approved the submitted version.

## Conflict of Interest

The authors declare that the research was conducted in the absence of any commercial or financial relationships that could be construed as a potential conflict of interest.
